# Comment on Tomaskova et al. Mortality in Miners with Coal-Workers’ Pneumoconiosis in the Czech Republic in the Period 1992–2013. *Int. J. Environ. Res. Public Health*, 2017, *14*, 269

**DOI:** 10.3390/ijerph15020276

**Published:** 2018-02-06

**Authors:** Mei Yong

**Affiliations:** Institute for Occupational Epidemiology and Risk Assessment (IERA), Evonik Industries AG, Rellinghauser Straße 1-11, 45128 Essen, Germany; mei.yong@evonik.com; Tel.: +49-201-177-4400; Fax: +49-201-177-4403

**Keywords:** coal workers’ pneumoconiosis (CWP), lung cancer

## Abstract

With interest, I read the recent analysis by Tomaskova and co-workers (2017) about mortality from coal workers’ pneumoconiosis (CWP). The research question remains unclear whether coal workers’ pneumoconiosis (CWP) resulting from exposure to respirable coal dust containing crystalline silica accelerates the development of lung cancer or whether it is an intermediate stage in the pathway. I made several points of considerations with respect to (1) qualified data; (2) alternate measures for excessive risks; and (3) methodological flaws that should be avoided.

## 1. Introduction

Tomaskova et al. [1] compared the total mortality and cause-specific mortality, and lung cancer risk in particular, among coal miners with acknowledged coal workers’ pneumoconiosis (CWP), and without CWP in the Czech Republic in the period 1992–2013. The authors found that the total mortality of the first cohort of 3476 coal miners with CWP (SMR = 1.10, 95% CI: 1.02–1.17) was significantly elevated in comparison to that of the second cohort of 6687 former coal miners (SMR = 0.86, 95% CI: 0.82–0.91) who were removed due to the achieved maximal permissible exposure (MPE), and were free of CWP through 2013. Within each cohort, SMRs were calculated using the mortality risks of the general population as reference.

Interestingly, the mean age at death for those coal miners with CWP from diseases of the respiratory system was 70.5 (SD: 10.9), while those without CWP died at 61.1 (SD: 8.5). The longer lifetime of miners with CWP seemed to be contradictory to the elevated SMRs. Nonetheless, the authors drew their conclusion that the measure of the MPE prevents CWP or allows only a mild form of the disease and, thus, reduces the mortality of coal miners to the level of the general population. This conclusion seems to be illogical because the cohort with CWP must have shared the same health protection measures.

In fact, the authors addressed an important research question, of whether CWP is a marker or an intermediate of lung cancer. However, the answer remains unclear. The rationale of this research is that high level of coal dust exposure may lead to chronic inflammation, which consequently results in fibrosis e.g., pneumoconiosis and finally develops into lung cancer. However, the information on exposure level and the comparability in terms of exposure between both cohorts was not provided in Tomaskova et al. [1].

I reviewed the study of Tomaskova et al. [1], and made several points of considerations with respect to (1) qualified data; (2) alternate measures for excessive risks; and (3) methodological flaws which should be addressed.

## 2. Qualified Data Needed

The complete pathway to address this research question consists of exposure to dust, risk of developing CWP, and finally lung cancer. Accordingly, we need the information on (1) exposure measurements; (2) diagnosis of CWP; and (3) diagnosis of cancer. First, Tomaskova et al. [1], did not provide the information on dust exposure and potential confounding factors. Therefore, this research is not able to answer the basic question of whether the increased lung cancer risk is attributable to coal dust. Results from literature with respect to this point are also still inconsistent. Most of the cohort studies failed to report an increased risk of lung cancer. In addition, the comparability of CWP and the non-CWP states is a fundamental requirement to compare the accelerating risk of developing lung cancer while the non-CWP of the control cohort is a state of fiction. Tomaskova et al. [1] used maximal permissible exposure (MPE) as a proxy of exposure for the non-CWP cohort, while the exposure level of the CWP cohort remained unknown. Based on the study reported before [2], the comparability in terms of exposure duration, concentration, and cumulative concentration remains untestable because of the inherent limitation of the study.

Secondly, determination of CWP was based on the National Registry of Occupational Diseases. The reliability of diagnosis from this source is rarely tested. In a study by the German porcelain industry cohort, in which morbidity of silicosis was investigated [3], relevant overestimation was demonstrated after a comparison of the original classification based on the data from the employer’s liability insurance association to the diagnosis of silicosis by re-reading all radiographs, retrospectively, by two independent readers [3]. Because the routine radiographic readings were intended for prevention (i.e., early detection of radiographic changes consistent with pneumoconiosis) as well as administrative purposes (i.e., workers’ compensation) and not for etiological research purposes, a misclassification of CWP is possible.

Finally, ascertainment of causes of death was based on death certificate only (DCO), which is a commonly used data quality indicator. The diagnoses notified, however, may arise from various sources during the lifetime of the individuals. Commonly, a mono-causal concept, namely the underlying cause of death is determined and coded as a single cause of death for statistical purposes, may not be fully in accordance with the multifactorial death process [4]. It is suspected that physicians are more prone to identify the respiratory diseases, in particular CWP or lung cancer to be the cause of death for coal miners than for general population [3]. Hence, excess risk tends to be overestimated if comparing to general population. A complete follow-up of the health profiles of a well-defined cohort along with exposure measurements at an individual level would be qualified to address this question.

## 3. Alternate Measures of Excessive Risk

Tomaskova et al. [1] used age-standardized mortality ratios (SMR) to estimate the relative risk in the respective cohort of coal miners with and without CWP. SMR measures excessive risk, correcting for age, sex, and calendar year, in comparison to the general population. However, the distortion of risk estimates because of smoking, which is the most relevant confounding factor, is not considered. Several approaches, such as Axelson’s approach [5] and Bayesian bias adjusting approach [6] are available to derive a valid measure. Furthermore, a ratio of the SMRs of both cohorts, which can be computed according to Newcomber and Altman [7], would make a direct comparison between the two cohorts possible. Alternative measure, such as expected year of life lost (e-YLL) suggested by Park et al. [8] and extensively discussed by Morfeld [9], would be particularly useful for this study.

## 4. Methodological Considerations

In addition to confounding by smoking and other potential hazardous substances, retrospective design, and incomplete information on exposure assessment may hamper Tomaskova et al. [1] and their ability to address the bias that occurs by time-related factors analytically: (i) time-since-hire effect; (ii) time-dependent exposure level: workers hired early tended to have higher exposure; and (iii) selection due to healthy hire effect and healthy survivor effect [10].

In particular, the healthy hire effect, i.e., selection of healthy individuals into the occupational cohort at time of hire so that their disease risks differ from the disease risks in the source (general) population. Furthermore, healthy survivor effect, i.e., miners with CWP or miners early stage findings were likely to quit earlier than those without CWP because of different health statuses, introducing additional study bias.

In summary, to address this important question of whether CWP would accelerate the risk of developing lung cancer, information covering the whole pathway would be necessary, from exposure measurement of coal dust including crystalline silica, the time it takes to develop CWP, and consequently, the time to develop lung cancer and die from it.

The present study is apparently not able to distinguish the true association from the distortion of bias because of some severe inherent shortcomings, such as retrospective design, lack of information on exposure of interest, potential confounding factors, and untestable assumptions that would warrant the comparability between the cohorts.
